# Increased risk of deep vein thrombosis, pulmonary embolism, and all-cause mortality in chronic venous disorder: a large-scale retrospective cohort study

**DOI:** 10.3389/fmed.2025.1683970

**Published:** 2025-10-08

**Authors:** Eva Lotta Moderegger, Sören Dräger, Sophie L. Preuss, Artem Vorobyev, Patrick Terheyden, Khalaf Kridin, Katja Bieber, Ralf J. Ludwig, Birgit Kahle, Philip Curman

**Affiliations:** ^1^Department of Dermatology, University Clinic of Schleswig Holstein, Campus Lübeck, Lübeck, Germany; ^2^Lübeck Institute of Experimental Dermatology, University of Lübeck, Lübeck, Germany; ^3^Azrieli Faculty of Medicine, Bar-Ilan University, Safed, Israel; ^4^Unit of Dermatology and Skin Research Laboratory, Galilee Medical Center, Nahariya, Israel; ^5^Dermato-Venereology Clinic, Karolinska University Hospital, Stockholm, Sweden; ^6^Department of Medical Epidemiology and Biostatistics, Karolinska Institutet, Stockholm, Sweden; ^7^Dermatology and Venereology Division, Department of Medicine (Solna), Karolinska Institutet, Stockholm, Sweden

**Keywords:** chronic venous disorder, chronic venous insufficiency, chronic venous disease, varicose veins, pulmonary embolism, thrombosis, mortality, TriNetX

## Abstract

**Background:**

Chronic venous disorder (CVD), often overlooked as a significant medical burden, has recently been linked to severe health risks, especially deep vein thrombosis (DVT), and pulmonary embolism (PE). However, large-scale data are lacking. Specifically, the impact of CVD severity on the risk of thromboembolic events and the impact of procedural interventions on these risks are unknown.

**Methods:**

A retrospective cohort study of mortality and serious adverse events was conducted using electronic health records derived from the TriNetX database. Propensity-score matching and sensitivity analyses were performed to mitigate bias.

**Results:**

We included 463,313 patients with CVD. An increased risk of superficial vein thrombosis [SVT; hazard ratio (HR), 19.0, 95% confidence interval (CI) 17.1–21.0, *p* < 0.0001], DVT (3.3, 3.2–3.6), PE (2.1, 2–2.1), and mortality (1.8, 1.8–1.8) were observed. These results persisted in two sensitivity analyses. When stratifying CVD for disease severity into chronic venous disease and -insufficiency, elevated risks of thromboembolic events and all-cause mortality were observed in both groups. Comparing CVD patients with interventions to those without, the risk of DVT (0.9, 0.8–0.9), PE (0.6, 0.5–0.6) and all-cause mortality (0.5, 0.5–0.5) decreased. Conversely, the risk of SVT increased (1.8, 1.6–2.0).

**Discussion:**

Independently of disease severity, CVD entails an increased risk for venous thromboembolic events and all-cause mortality. In CVD patients, procedural interventions are associated with reduced risks for DVT, PE and all-cause mortality. Confirmation of these potentially clinically relevant findings necessitates prospective randomized trials.

## 1 Introduction

Chronic venous disorder (CVD) affect a substantial proportion of the adult population (1, 2). These include chronic venous disease (CVDis, clinical stage C0–C2) and chronic venous insufficiency (CVI, C3–C6) (3). In Germany, it has been reported that ~23% of the adults, meaning every fifth woman and every sixth man, is affected by CVD (4). While they were generally believed not to pose severe health risks on their own (5), recent studies have associated CVD to potentially life-threatening diseases, specifically deep vein thrombosis (DVT) and pulmonary embolism (PE) (6–8).

In support of the latter notion, a retrospective cohort study based on 212,984 electronic health records (EHRs) from Taiwan's National Health Insurance program reported a significantly increased risk of DVT and PE in patients with varicose veins (8), which was attributed to a proinflammatory state in CVD with increased proinflammatory and prothrombotic markers. Similarly, a strong association of CVD and thrombosis was observed in the elderly (6). Furthermore, a causal effect of CVD on thrombosis has been proposed in a recent Mendelian randomization study (9). These findings indicate that CVD potentially impose serious health risks that may be greater than previously thought. However, studies taking potentially important confounding factors into account, such as large-scale matched cohort studies, are lacking. It furthermore remains uncertain whether the severity of CVD affects the risk of thromboembolic events and all-cause mortality, as well as whether procedural interventions could alter the risk of thromboembolic events and all-cause mortality.

To address these knowledge gaps, we conducted a large-scale propensity-score matched cohort study on 442,057 EHRs from US patients. Our objective was to evaluate the risks of superficial vein thrombosis (SVT), DVT, PE, and mortality in individuals with CVD. We further compared these risks between patients with CVDis and CVI as well as matched controls. Additionally, we conducted a time-restricted analysis and assessed the potential influence of CVD interventions on these outcomes.

## 2 Materials and methods

### 2.1 Ethics

This retrospective study is exempt from informed consent. The data reviewed is a secondary analysis of existing data, does not involve intervention or interaction with human subjects, and is de-identified per the de-identification standard defined in Section §164.514(a) of the HIPAA Privacy Rule. The process by which the data is de-identified is attested to through a formal determination by a qualified expert as defined in Section §164.514(b)(1) of the HIPAA Privacy Rule. This formal determination by a qualified expert was refreshed in December 2020.

### 2.2 Study design and data source

**Figure 1:**
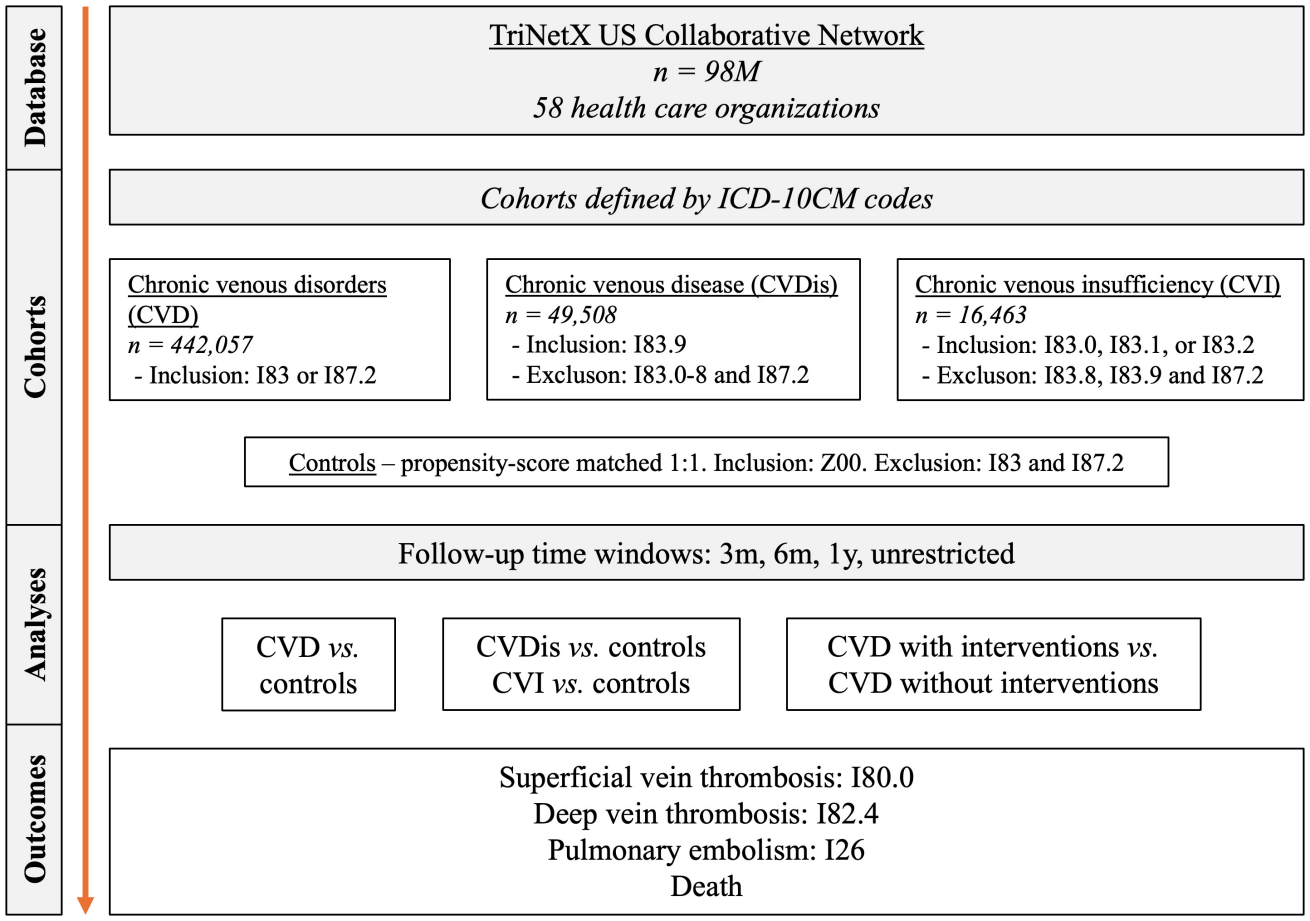
Study flow chart. CVD, chronic venous disorder; CVDis, chronic venous disease; CVI, chronic venous insufficiency; ICD-10CM, international classification of diseases, 10^th^ edition, clinical modification.

**Figure 2:**
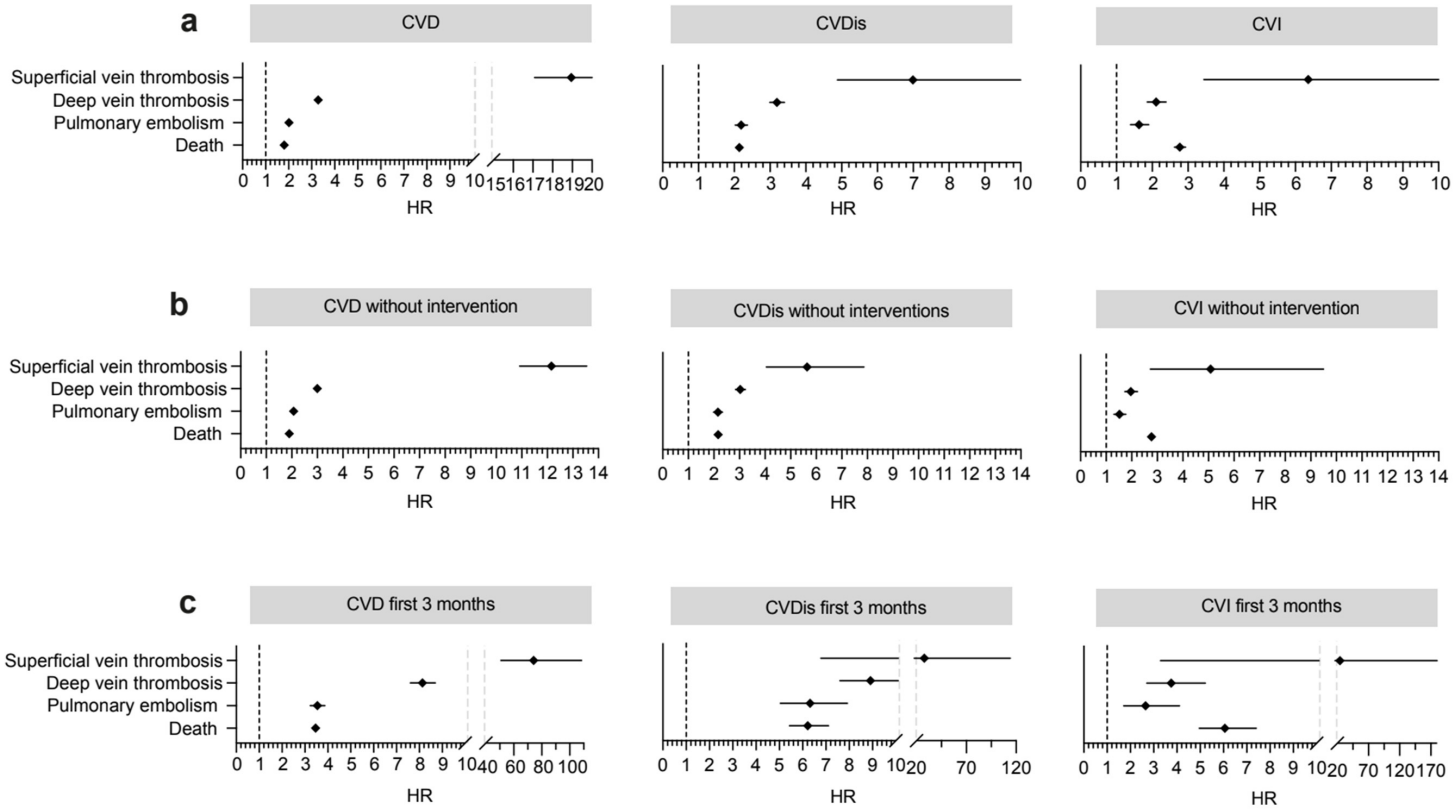
Outcomes for the chronic venous disorder (CVD) cohort, as well as chronic venous disease (CVDis) and chronic venous insufficiency (CVI) from 1 day to any time after index **(a)** and the same cohorts but excluding all interventions **(b)**. Results for the first 3 months after index are shown in the subfigure **(c)**.

**Figure 3:**
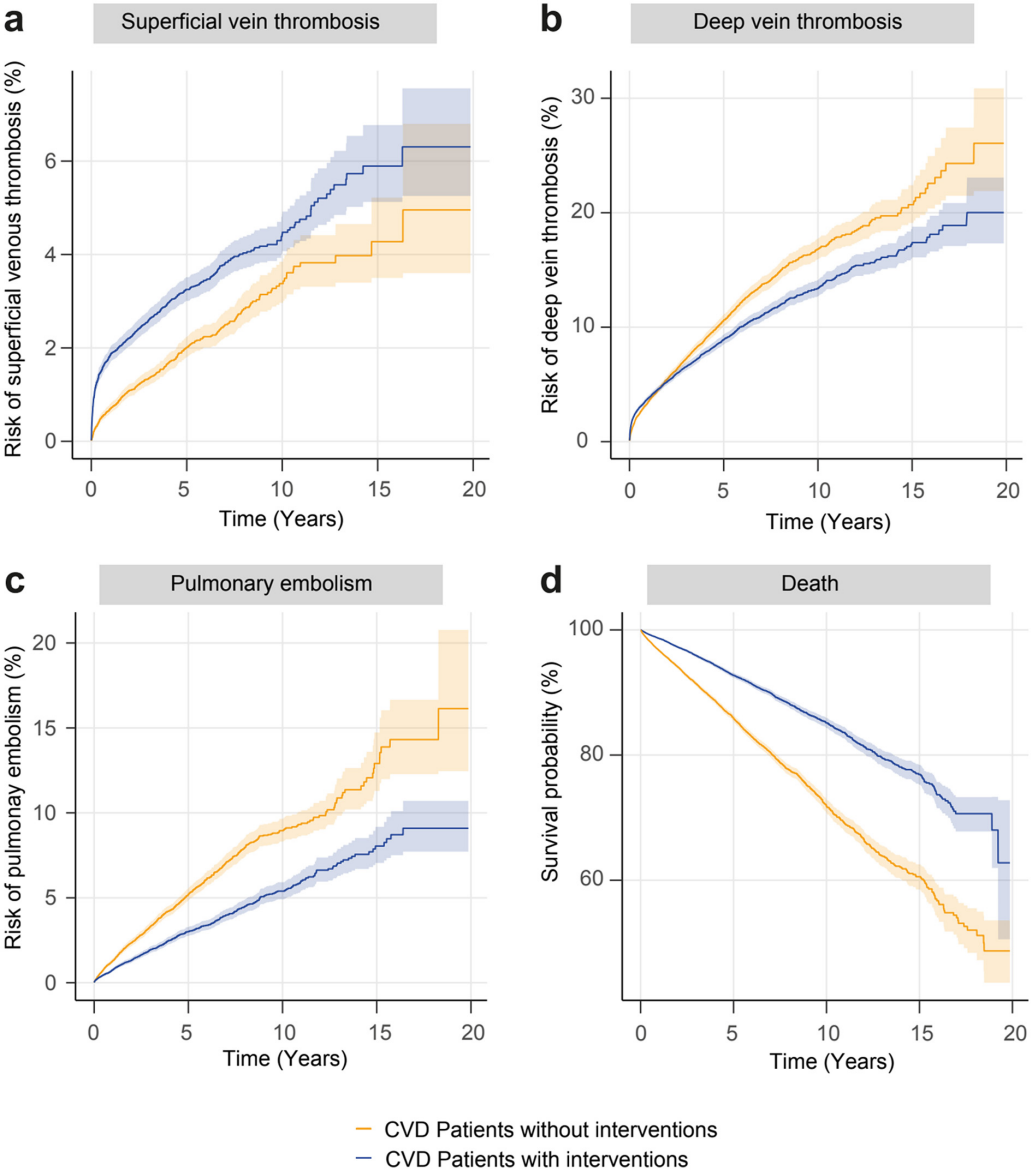
Nelson-Aalen plots **(a–c)** of the study outcomes and Kaplan-Meier plot **(d)** of the endpoint all-cause mortality in interventional chronic venous disorder (blue line) and non-interventional chronic venous disorder (orange line). All results are significant (*p*-value < 0.0001). CVD, chronic venous disorder.

A population-based retrospective cohort study with propensity-score matching (PSM) was performed using the US Collaborative Network of TriNetX following previously published protocols (10–12). TriNetX, LLC, provides a global data and analytics platform encompassing over 150 million EHRs (13). As part of a collaboration between the University medical hospital of Schleswig-Holstein (UKSH) and TriNetX, UKSH researchers have access to the TriNetX network. In this study, EHRs were retrieved from the US Collaborative Network that at the time of analysis included over 98 million EHRs from 58 Health Care Organizations (HCOs; Figure 1). Two main cohorts were retrieved: (1) patients with CVD and (2) control subjects without CVD (Supplementary Table 1). The controls were matched with each patient cohort and the risks of SVT, DVT, PE, and all-cause mortality were contrasted between the cohorts. Consequently, further analysis was conducted. Next, we included disease severity to contrast CVDis and CVI (Figure 2a). While CVDis and CVI are normally defined by CEAP clinical staging, ICD-10CM codes were utilized as proxys to define these subgroups to be viewed as a stratification for disease severity (Supplementary Table 1). To validate these findings, we performed two additional analyses: first, using an alternative definition of CVD and controls while excluding all interventions (Figure 2b); and second, by restricting the time window to 3 months (Figure 2c). We then directly compared the risk of SVT, DVT, PE and all-cause mortality in patients with CVD who underwent interventions (e.g., injection of sclerosant, ligation, endovenous ablation or stab phlebectomy) vs. those who did not (Figure 3). Interventional CVD was defined by the inclusion of any CPT code describing a CVD intervention after CVD diagnosis, while non-interventional CVD was defined by exclusion of the same codes (Supplementary Table 2). In all subgroup cohorts the inclusion code had to occur twice at least 3 months apart.

### 2.3 Study population

The study period was August 2003 to December 2023, with variable individual entry points for participants. The following criteria were used to define the cohorts: the cohorts were defined by EHRs with codes listed in Supplementary Table 1. To increase the validity of the diagnosis, either code had to be coded twice. Controls were defined as individuals with a code of general medical encounter, while excluding the CVD codes. To ensure follow-up and allow PSM for 13 variables these criteria had to be met at two independent visits at least 12 months apart.

### 2.4 Outcomes

The following outcomes were used: SVT (ICD-10CM:I80.0), DVT (ICD-10CM:I82.4), PE (ICD-10CM:I26), and all-cause mortality. Outcomes were defined prior to data analysis.

### 2.5 Covariates

PSM between cases and controls was implemented as a measure against bias. Covariates included in all analyses were: age at index (continuous variable), female sex (binary), essential hypertension (ICD-10CM:I10, binary), disorders of lipoprotein metabolism and other lipidemias (ICD-10CM:E78, binary), neoplasms (ICD-10CM:C00-D49, binary), diabetes (ICD-10CM:E08-E13, binary), ischemic heart diseases (ICD-10CM:I20-I25, binary), chronic kidney disease (ICD-10CM:I18), nicotine dependence (ICD-10CM:F17, binary), any surgical procedure (CPT 1003143, binary), hospital inpatient service (CPT 1013659, binary), systemic contraceptive (HS200, binary), and body mass index (BMI, TNX curated 9083, continuous variable). In a sensitivity analysis with extended PSM (see below) the additional covariate was included in the PSM: personal history of venous thrombosis and embolism (ICD-10CM:Z86.71). A propensity-score for each patient was generated by logistic regression using the Python package *Scikit-learn*. Matching was performed 1:1 using the greedy nearest neighbor approach with a cut-off distance of 0.1 pooled standard deviations of the logit of the propensity-score. Baseline characteristics were re-evaluated and reported after matching, differences were compared by *t*-test for continuous and z-test for binary or categorical variables.

### 2.6 Primary, sensitivity and subgroup analyses

Outcomes for all analyses were investigated for several time windows: 1 day to 3 months, 6 months, 12 months, and any time after index. Furthermore, three sensitivity analyses were conducted: (1) alternative definition of the CVD with the exclusion of all interventions (Figure 2b, Supplementary Table 2), (2) extended PSM (Supplementary Table 3), and (3) outcomes 1 year to any time after index. To mitigate detection bias, outcomes prior to index were excluded for all analyses (Table 1).

**Table 1:** Study outcomes in patients with chronic venous disorder.

**Time restriction**	**Patients with chronic venous disorder**			**Controls**			**Statistics**		
	***N*** **of eligible participants**	***N*** **of outcomes**	**Risk (%)**	***N*** **of eligible participants**	***N*** **of outcomes**	**Risk (%)**	**Risk, difference (95% CI) (%)**	**HR** **(95% CI)**	***P*****-value (**α_adj_ = **0.0125, log-rank)**
**Superficial vein thrombosis**									
1 day to any time	432,960	6,931	1.601	441,510	378	0.086	1.515 (1.477, 1.554)	18.945 (17.082, 21.012)	<0.0001
1 day to 3 months	450,009	1,943	0.432	458,846	27	0.006	0.426 (0.407, 0.445)	74.184 (50.74, 108.46)	<0.0001
1 day to 6 months	450,009	2,801	0.622	458,846	57	0.012	0.61 (0.587, 0.633)	51.142 (39.345, 66.476)	<0.0001
1 day to 12 months	450,009	3,703	0.823	458,846	105	0.023	0.8 (0.773, 0.827)	37.053 (30.52, 44.984)	<0.0001
1 year to any time	469,550	3,529	0.752	482,489	314	0.065	0.686 (0.661, 0.712)	11.507 (10.252, 12.915)	<0.0001
**Deep vein thrombosis**									
1 day to any time	399,121	29,456	7.38	427,164	9,860	2.308	5.072 4.979, 5.165	3.283 3.209, 3.359	<0.0001
1 day to 3 months	414,611	7,202	1.737	443,145	962	0.217	1.52 (1.478, 1.562)	8.133 (7.604, 8.699)	<0.0001
1 day to 6 months	414,611	10,151	2.448	443,145	1,666	0.376	2.072 (2.022, 2.123)	6.698 (6.36, 7.054)	<0.0001
1 day to 12 months	414,611	13,815	3.332	443,145	2,928	0.661	2.671 (2.612, 2.731)	5.261 (5.056, 5.476)	<0.0001
1 year to any time	422,481	17,275	4.089	463,575	7,847	1.693	2.396 (2.326,2.467)	2.409 (2.345, 2.474)	<0.0001
**Pulmonary embolism**									
1 day to any time	422,865	13,824	3.269	432,119	7,012	1.623	1.646 (1.581, 1.712)	2.017 (1.96, 2.076)	<0.0001
1 day to 3 months	439,514	2,079	0.473	448,999	607	0.135	0.338 (0.315, 0.361)	3.54 (3.234, 3.875)	<0.0001
1 day to 6 months	439,514	3,091	0.703	448,999	1,103	0.246	0.458 (0.429, 0.486)	2.926 (2.732, 3.135)	<0.0001
1 day to 12 months	439,514	4,771	1.086	448,999	1,980	0.441	0.645 (0.608, 0.681)	2.55 (2.42, 2.687)	<0.0001
1 year to any time	457,700	9,744	2.129	470,546	5,421	1.152	0.977 (0.925, 1.029)	1.828 (1.768, 1.89)	<0.0001
**Deceased**									
1 day to any time	439,431	64,075	14.581	439,245	34,926	7.951	6.63 6.498, 6.761	1.806 (1.783, 1.83)	<0.0001
1 day to 3 months	456,786	7,798	1.707	456,707	2,278	0.499	1.208 (1.166, 1.251)	3.461 (3.303, 3.626)	<0.0001
1 day to 6 months	456,786	12,522	2.741	456,707	4,379	0.959	1.783 (1.727, 1.838)	2.922 (2.824, 3.025)	<0.0001
1 day to 12 months	456,786	19,832	4.342	456,707	8,391	1.837	2.504 (2.434, 2.575)	2.446 (2.384, 2.509)	<0.0001
1 year to any time	460,118	48,217	10.479	471,843	28,961	6.138	4.341 (4.23, 4.453)	1.632 (1.609, 1.656)	<0.0001

The risk of superficial vein thrombosis, deep vein thrombosis, pulmonary embolism, and all-cause mortality in individuals patients with chronic venous disorder for all different study time windows.

CI, confidence interval; HR, hazard ratio.

### 2.7 Statistical analysis

Relative risks and risk differences were calculated. Survival analyses were performed using the Kaplan-Meier (KM) method. KM curves were compared using the Log-rank test; *p*-values of <0.05 were considered significant. To correct for multiple testing bias, Bonferroni correction was used (α_adjust_ = 0.0125) for the four outcomes of interest. Nelson-Aalen plots were utilized to test the proportionality assumption. A univariate Cox proportional hazards regression was used to express hazard ratios (HRs). All statistical analyses, with the exception for Bonferroni correction, were conducted on the TriNetX platform.

### 2.8 Use of artificial intelligence

ChatGPT-4o (OpenAI LCC, San Francisco, California, USA) was used to extract raw data to table format and improving readability and language of sections of the manuscript. All extracted data and revisions were thoroughly reviewed and validated by the authors. The authors take full responsibility for the accuracy, integrity, and final content of the manuscript.

## 3 Results

### 3.1 Cohort description and patient characteristics

Data from just over 98 million individuals was sampled. After successful PSM, 442,057 patients (62.1 % females) with CVD, 49,508 patients with CVDis, and 16,463 patients with CVI were retrieved, with equally sized control groups (Figure 1). Baseline characteristics for the total CVD cohort and controls are detailed in Table 2. In all cohorts, a female predominance was present. No major differences were seen in any of the matching covariates following PSM.

**Table 2:** Baseline characteristics before and after propensity score matching in patients with chronic venous disorder.

**Characteristic**	**Before matching**		**After matching**	
	**Patients with chronic venous disorder**	**Controls**	**Patients with chronic venous disorder**	**Controls**
Number of participants	463,313	6,175,166	442,057	442,057
Age at Index (years, SD)	61.7 ± 16.2	32.7 ± 25.9	61.7 ± 16.2	61.8 ± 16.2
Female (%)	62.1	54.6%	62.1	62.4
BMI	31.1 +/– 7.6	24.1 +/– 7.7	31.1 +/– 7.6	29.0 +/– 6.6
Primary hypertension (%)	53.7	22.6	53.7	53.9
Dyslipoproteinemia (%)	45.7	24.6	45.7	46.0
Neoplasms (%)	36.6	17.6	36.6	36.7
Diabetes mellitus (%)	28.4	8.8	28.4	28.2
Ischemic heart diseases (%)	21.4	5.2	21.4	21.3
Chronic kidney disease (%)	15.7	3.8	15.7	15.3
Nicotine dependence (%)	13.7	6.4	13.7	13.6
Surgery (%)	61.2	43.8	61.2	61.8
Hospital inpatient services (%)	19.7	6.5	19.7	19.5
Contraceptives, systemic (%)	3.9	5.5	3.9	3.8

SD, standard deviation; Std. diff., standardized difference.

### 3.2 Increased risks of SVT, DVT, PE, and all-cause mortality in CVD

Increased risks for all study outcomes were found for all time windows, with the primary analysis of outcomes 1 day to any time after index showing risk elevations for SVT by 18.9 times [hazard ratio (HR) 18.95, confidence interval (CI) 17.08–21.01, *p* < 0.0001], DVT by 3.3 times (HR 3.28, CI 3.21–3.36), PE by 2.0 times (HR 2.02, CI 1.96–2.08), and all-cause mortality by 1.8 times (HR 1.81, CI 1.78–1.83, *p* < 0.0001; Figure 2a, Table 1). Increased risks were also seen in the CVDis and CVI subgroups for all outcomes (Figure 2a), i.e., disease severity did not greatly affect the risk of adverse events. Furthermore, the significant risk elevations persisted when excluding interventions for both the CVD, CVDis, and CVI cohort definitions (Figure 2b).

The most pronounced risks were observed during the 3-month follow-up: the risk of SVT was elevated by 74 times (HR, 74.18, 95% CI 50.74–108.46, *p* < 0.0001), DVT by 8 times (HR 8.13, CI 7.60–8.70, *p* < 0.0001), PE by 3.5 times (HR 3.54, CI 3.23–3.88, *p* < 0.0001), and all-cause mortality by 3.5 times (HR 3.46, CI 3.30–3.63, *p* < 0.0001; Figure 2c, Table 1). Extending the PSM by including personal history of venous thrombosis and embolism did not considerably alter the results (Supplementary Table 3). For absolute risks please refer to Table 1.

### 3.3 Increased risks for adverse events in interventional compared to non-interventional CVD

When comparing patients with CVD having undergone procedural interventions to those without any interventions, the risks of both DVT, PE, and all-cause mortality were significantly reduced, while the risk of SVT was significantly elevated (Figure 3, Supplementary Table 4). Comparable results were seen when performing the same analysis with extended PSM (results not shown).

## 4 Discussion

We found an increased risk of SVT, DVT, PE, and all-cause mortality in patients with CVD, which was also present for both CVDis and CVI. The reason behind this might be increased proinflammatory and prothrombotic factors in the circulation, as was noted by several other studies. The highest risks were observed in close association to first diagnosis. Procedural interventions were associated with an elevated risk of SVT, but importantly with decreased risks of DVT, PE, and all-cause mortality.

Previous studies have also observed an increased risk of DVT (6–8, 14) and PE (6, 8) in patients with CVD. Li et al. found a causal association between genetically predicted varicose veins and DVT in a Mendelian randomization study (9), and the Gutenberg Health study reported and increased risk of cardiovascular disaease and all-cause mortality in patients with CVDis (20). However, a comprehensive insight into all investigated endpoints had been missing. Furthermore, the sample size of previous studies is relatively low compared to the present investigation. Moreover, we included nicotine dependence and contraceptives in the PSM, both important risk factors of DVT and PE (15, 16).

As a clinically relevant extension to the previous studies, we also investigated the impact of disease severity on thromboembolic events and all-cause mortality. CVDis and CVI both conveyed an increased risk of DVT, PE and all-cause mortality, with the latter being slightly increased in CVI compared to CVDis.

In addition, our analysis provides a detailed insight into the risk for venous thromboembolic events and all-cause mortality during the first year after initial diagnosis, and the first 3 months in particular, since patients with CVD exhibited the highest risk of the adverse events during the first 3 months after the diagnosis. When we restricted the follow-up analysis from 1 year to any time after diagnosis, the risk of all outcomes persisted but were attenuated. To exclude that these results are related to patients who underwent interventions, we repeated the same time restriction analysis but excluded patients who underwent interventions in the respective cohorts and matched controls. Only the risk of SVT decreased, indicating that interventions may lead to SVT, while the risks of the other outcomes are not affected. These discoveries carry implications for the care of patients with CVD, emphasizing the necessity for heightened surveillance during the initial year following diagnosis, aiming at increased patient and caretaker awareness.

To date, no studies addressing the impact of interventions in patients with CVD on thromboembolic events and all-cause mortality exists. Analyzing the impact of interventions revealed an intervention-associated reduced risk of DVT, PE and all-cause mortality. However, these interventions were associated with a slightly increased risk of SVT, which is in line with current data (17). These findings warrant further investigations into the optimal timing of interventions, since in the present study, thromboembolic events peaked during the first 3 months after diagnosis.

This study has several strengths. First, it leverages a large-scale population-based cohort from the TriNetX network, which includes over 98 million individuals, providing a robust dataset for assessing the relationship between CVD and thromboembolic events. The use of PSM helps mitigate confounding, enhancing the validity of our findings. Additionally, this study is one of the first to investigate the impact of procedural interventions on the risk of adverse outcomes in patients with CVD, offering clinically relevant insights for managing this condition. Nevertheless, we did not contrast the different interventions with one another. However, several limitations must be acknowledged: the retrospective nature of the study, relying on EHRs, is susceptible to potential miscoding, particularly in distinguishing between CVDis and CVI. Furthermore, the absence of clinical classifications like CEAP in the database limited our ability to assess disease severity comprehensively and define cohorts accordingly. The diagnoses were based on coding, which may not capture the entire patient history, especially for events occurring outside the contributing HCOs. The lack of an ICD-10CM-code for family history of venous thrombosis, a recognized risk factor for venous thromboembolism (18, 19), prevented its inclusion in the PSM process. Finally, while our findings suggest associations, the observational design does not allow for causal inference.

In conclusion, our study demonstrates a clear increase in the risk of venous thromboembolic events and mortality in patients with CVD, regardless of the disease severity. These findings underscore the importance of recognizing CVD as a significant risk factor for thromboembolic events, advocating for its inclusion in clinical risk assessment models. Furthermore, the observed association between procedural interventions and a reduced risk of DVT, PE and mortality highlights the potential benefit of timely interventions in the management of CVD. Incorporating these insights into clinical practice may improve patient outcomes, emphasizing the need for proactive management strategies in patient care.

## Data Availability

The raw data supporting the conclusions of this article will be made available by the authors, without undue reservation.
