# Supplementary material for: Increased risk of deep vein thrombosis, pulmonary embolism, and all-cause mortality in chronic venous disorder: a large-scale retrospective cohort study
Source: Front Med (Lausanne). 2025 Oct 8;12:1683970. doi: 10.3389/fmed.2025.1683970 (PMC12540070; doi:10.3389/fmed.2025.1683970)
Supplement: Supplementary file 1 [file Data_Sheet_1.docx]

Supplementary Material

# Supplementary tables

## Supplementary table 1. Detailed cohort definitions for all cohorts using the International Classification of Diseases 10^th^ edition Clinical Codification (ICD-10CM).

| Chronic venous disorder (CVD) | Controls |
| --- | --- |
| *Inclusion criteria*  Varicose veins of lower extremities (I83) or Venous insufficiency, chronic, peripheral (I87.2).  *The inclusion criteria had to be coded twice at least three months apart.* | *Inclusion criteria*  Encounter for general examination without complaint, suspected or reported diagnosis (Z00).  *Exclusion criteria*  Varicose veins of lower extremities (I83) or Venous insufficiency, chronic, peripheral (I87.2).  *The inclusion criteria had to be coded twice at least 12 months apart.* |
| Chronic venous disease (CVDis) |  |
| *Inclusion criteria*  Asymptomatic varicose veins of lower extremities (I83.9).  *Exclusion criteria*  Varicose veins of lower extremities with either ulcer (I83.0), inflammation (I83.1), both ulcer and inflammation (I83.2), or other complications (I83.8); or venous insufficiency, chronic, peripheral (I87.2).  *The inclusion criteria had to be coded twice at least three months apart.* |  |
| Chronic venous insufficiency (CVI) |  |
| *Inclusion criteria*  Varicose veins of lower extremities with either ulcer (I83.0), inflammation (I83.1), or both ulcer and inflammation (I83.2).  *Exclusion criteria*  Varicose veins of lower extremities with other complications (I83.8), asymptomatic varicose veins of lower extremities (I83.9), or venous insufficiency, chronic, peripheral (I87.2).  *The inclusion criteria had to be coded twice at least three months apart.* |  |

## Supplementary table 2. Interventions for chronic venous disorder with their respective Current Procedural Terminology (CPT) codes.

| **CPT code** | **Procedure** |
| --- | --- |
| 36468 | Injection(s) of sclerosant for spider veins (telangiectasia), limb or trunk |
| 36470 | Injection of sclerosant; single incompetent vein (other than telangiectasia) |
| 36471 | Injection of sclerosant; multiple incompetent veins (other than telangiectasia), same leg |
| 36473 | Endovenous ablation therapy of incompetent vein, extremity, inclusive of all imaging guidance and monitoring, percutaneous, mechanochemical; first vein treated |
| 36474 | Endovenous ablation therapy of incompetent vein, extremity, inclusive of all imaging guidance and monitoring, percutaneous, mechanochemical; subsequent vein(s) treated in a single extremity, each through separate access sites (List separately in addition to code for primary procedure) |
| 36475 | Endovenous ablation therapy of incompetent vein, extremity, inclusive of all imaging guidance and monitoring, percutaneous, radiofrequency; first vein treated |
| 36476 | Endovenous ablation therapy of incompetent vein, extremity, inclusive of all imaging guidance and monitoring, percutaneous, radiofrequency; subsequent vein(s) treated in a single extremity, each through separate access sites (List separately in addition to code for primary procedure) |
| 36478 | Endovenous ablation therapy of incompetent vein, extremity, inclusive of all imaging guidance and monitoring, percutaneous, laser; first vein treated |
| 36479 | Endovenous ablation therapy of incompetent vein, extremity, inclusive of all imaging guidance and monitoring, percutaneous, laser; subsequent vein(s) treated in a single extremity, each through separate access sites (List separately in addition to code for primary procedure) |
| 37241 | Vascular embolization or occlusion, inclusive of all radiological supervision and interpretation, intraprocedural roadmapping, and imaging guidance necessary to complete the intervention; venous, other than hemorrhage (eg, congenital or acquired venous malformations, venous and capillary hemangiomas, varices, varicoceles) |
| 37242 | Vascular embolization or occlusion, inclusive of all radiological supervision and interpretation, intraprocedural roadmapping, and imaging guidance necessary to complete the intervention; arterial, other than hemorrhage or tumor (eg, congenital or acquired arterial malformations, arteriovenous malformations, arteriovenous fistulas, aneurysms, pseudoaneurysms) |
| 37243 | Vascular embolization or occlusion, inclusive of all radiological supervision and interpretation, intraprocedural roadmapping, and imaging guidance necessary to complete the intervention; for tumors, organ ischemia, or infarction |
| 37244 | Vascular embolization or occlusion, inclusive of all radiological supervision and interpretation, intraprocedural roadmapping, and imaging guidance necessary to complete the intervention; for arterial or venous hemorrhage or lymphatic extravasation |
| 37700 | Ligation and division of long saphenous vein at saphenofemoral junction, or distal interruptions |
| 37718 | Ligation, division, and stripping, short saphenous vein |
| 37722 | Ligation, division, and stripping, long (greater) saphenous veins from saphenofemoral junction to knee or below |
| 37735 | Ligation and division and complete stripping of long or short saphenous veins with radical excision of ulcer and skin graft and/or interruption of communicating veins of lower leg, with excision of deep fascia |
| 37760 | Ligation of perforator veins, subfascial, radical (Linton type), including skin graft, when performed, open,1 leg |
| 37761 | Ligation of perforator vein(s), subfascial, open, including ultrasound guidance, when performed, 1 leg |
| 37780 | Ligation and division of short saphenous vein at saphenopopliteal junction (separate procedure) |
| 37785 | Ligation, division, and/or excision of varicose vein cluster(s), 1 leg |
| 1006834 | Stab phlebectomy of varicose veins, 1 extremity |

## Supplementary table 3. Study outcomes in patients with chronic venous disorder with extended propensity-score matching.

*Abbreviations: CI: confidence interval; HR: hazard ratio; ICD-10CM: international classification of diseases, 10^th^ edition, clinical modification.*

| **Outcome** | **ICD-10CM code** | **Chronic venous disorder** | | | **Controls** | | | **Statistics** | | |
| --- | --- | --- | --- | --- | --- | --- | --- | --- | --- | --- |
|  |  |  |  |  |  |  |  |  |  |  |
|  |  | **N of eligible participants** | **N of outcomes** | **Risk** (%) | **N of eligible participants** | **N of outcomes** | **Risk** (%) | **Risk, difference**  **(95% CI)** (%) | **HR**  **(95% CI)** | **P value**  (α_adj_=0.0125, log-rank) |
| 1 day to any time | | | | | | | | | | |
| **Superficial vein thrombosis** | I80.0 | 485,541 | 7,518 | 1.548 | 494,977 | 487 | 0.098 | 1.45  (1.414,1.486) | 16.331  (14.9,17.898) | < 0.0001 |
| **Deep vein thrombosis** | I82.4 | 444,537 | 33,972 | 7.642 | 471,556 | 12,456 | 2.641 | 5.001  (4.91,5.091) | 3.06  (2.998,3.123) | < 0.0001 |
| **Pulmonary embolism** | I26 | 472,034 | 16,760 | 3.551 | 478,695 | 9,180 | 1.918 | 1.633  (1.567,1.698) | 1.917  (1.869,1.966) | < 0.0001 |
| **Deceased** | | 491,694 | 74,821 | 15.217 | 491,690 | 43,186 | 8.783 | 6.434  (6.306,6.562) | 1.776  (1.755,1.797) | < 0.0001 |

## Supplementary table 4. Study outcomes in patients with chronic venous disorder having undergone procedural interventions compared to patients without any interventions.

*Abbreviations: CI: confidence interval; HR: hazard ratio; ICD-10CM: international classification of diseases, 10^th^ edition, clinical modification.*

| **Outcome** | **ICD-10CM code** | **Chronic venous disorder with interventions** | | | **Chronic venous disorder without interventions** | | | **Statistics** | | |
| --- | --- | --- | --- | --- | --- | --- | --- | --- | --- | --- |
|  |  |  |  |  |  |  |  |  |  |  |
|  |  | **N of eligible participants** | **N of outcomes** | **Risk** (%) | **N of eligible participants** | **N of outcomes** | **Risk** (%) | **Risk, difference**  **(95% CI)** (%) | **HR**  **(95% CI)** | **P value**  (α_adj_=0.0125, log-rank) |
| 1 day to any time | | | | | | | | | | |
| **Superficial vein thrombosis** | I80.0 | 28,579 | 764 | 2.673 | 29,802 | 438 | 1.47 | 1.204  (0.972,1.435) | 1.76  (1.565,1.979) | < 0.0001 |
| **Deep vein thrombosis** | I82.4 | 26,980 | 1,969 | 7.298 | 26,758 | 2,082 | 7.781 | -0.483  (-0.929,-0.036) | 0.871  (0.819,0.926) | < 0.0001 |
| **Pulmonary embolism** | I26 | 29,798 | 720 | 2.416 | 29,224 | 1,101 | 3.767 | -1.351  (-1.631,-1.072) | 0.58  (0.527,0.637) | < 0.0001 |
| **Deceased** | | 30,686 | 1,947 | 6.345 | 30,672 | 3,543 | 11.551 | -5.206  (-5.656,-4.756) | 0.487  (0.461,0.514) | < 0.0001 |
